# Interactive Maps to Improve Stroke Systems of Care in Wisconsin

**DOI:** 10.5888/pcd21.230166

**Published:** 2024-07-18

**Authors:** Ka Z. Xiong, Lena Swander, Dot Bluma, Joshua Tootoo, Marie Lynn Miranda, Melissa Fiffer

**Affiliations:** 1 Wisconsin Department of Health Services, Madison; 2 MetaStar, Madison, Wisconsin; 3 Children’s Environmental Health Initiative, University of Illinois Chicago; 4 Departments of Pediatrics and Mathematics, Statistics, and Computer Science, University of Illinois Chicago

**Figure:**
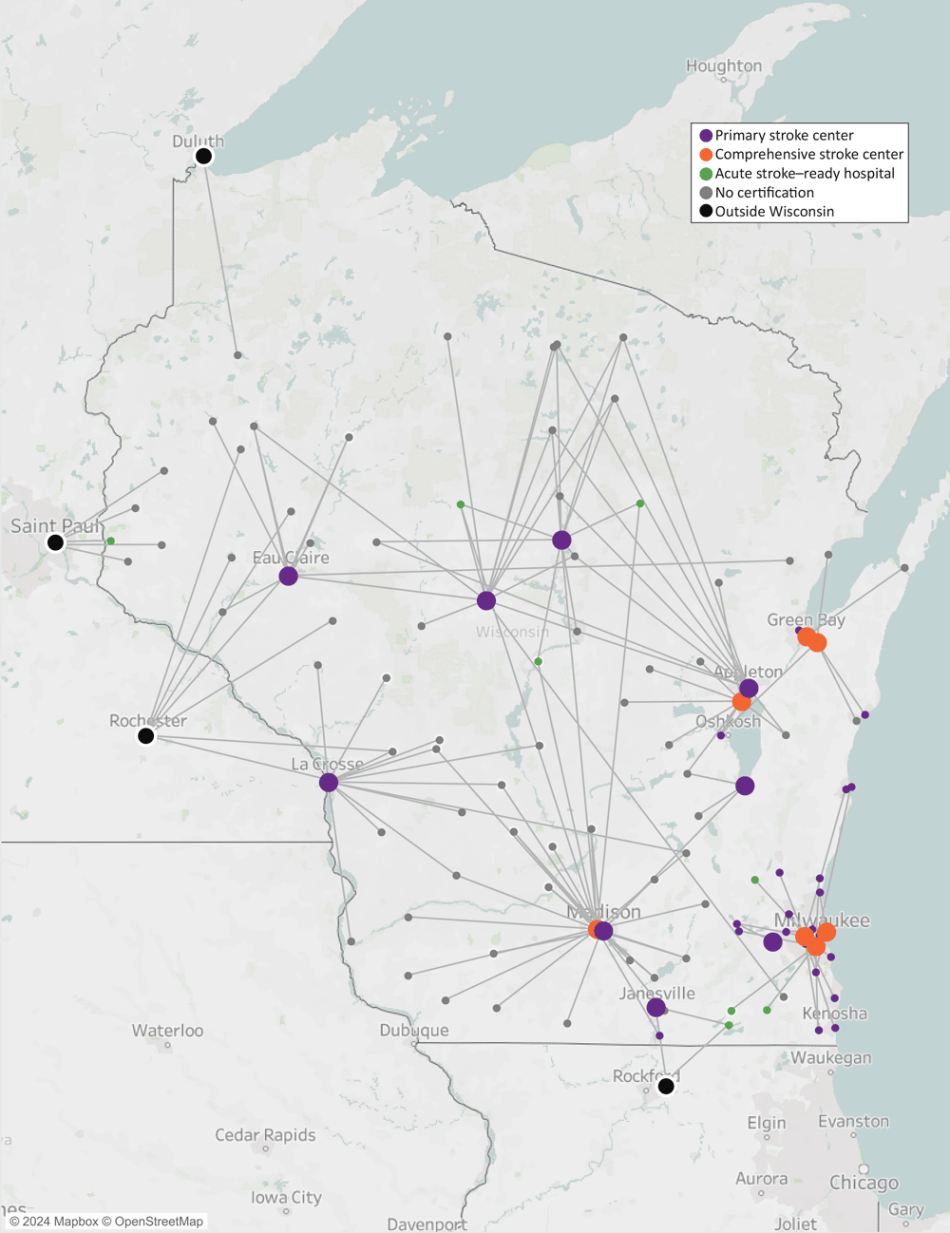
Transfer patterns for stroke patients from small rural hospitals (spokes, indicated by small circles) to larger tertiary hospitals (hubs, indicated by large circles) in Wisconsin. Visitors to the interactive map website (www.dhs.wisconsin.gov/coverdell/stroke-transfer-map.htm) can click on each transfer line to see estimated driving distance and time, along with the stroke certifications of hospitals. Transfer lines indicate driving distance between hospitals. Understanding the state’s stroke transfer patterns is important to improve care efficiency for emergency management services and hospitals. These maps play an important role in improving stroke systems of care in Wisconsin.

## Background

Stroke is the fifth leading cause of death in the US (1). In 2017, stroke accounted for more than 11,000 hospitalizations in Wisconsin (2). Minimizing the total time from onset of symptoms to delivery of treatment appropriate for the level of stroke complexity is critical to improving outcomes for stroke patients (3). Prehospital time — from recognition of symptoms to arrival at a local hospital — accounts for some delays in care. However, because patients are often transferred from local hospitals without substantial stroke-specific resources to larger hospitals capable of treating complex stroke patients, interhospital travel time is also important (4).

The Wisconsin Coverdell Stroke Program sought to learn more about barriers to ensuring that, when needed, stroke patients in all parts of the state receive advanced care without delay. As a partner in the CDC Paul Coverdell National Acute Stroke Program since 2012, The Wisconsin Coverdell Stroke Program aims to improve patient outcomes through stroke systems of care (5). The stroke systems of care framework entails increasing the quality of care and support for stroke patients across the continuum of care through close collaboration across emergency medical services agencies, hospitals, health care facilities, and statewide partners.

In 2020, with the help of the Get With The Guidelines–Stroke (GWTG) registry, the Wisconsin Coverdell Stroke Program determined that 24.6% of stroke patients in Wisconsin were transferred between hospitals to access advanced care or resources (6). In early 2021, the program administered surveys to 8 stroke coordinators across 5 public health regions to identify the facilities most frequently involved in stroke transfers (7). The program initially set out to create static maps depicting these transfers: one for the entire state and insets for 2 metropolitan areas (Milwaukee and Madison). During user testing, however, stroke coordinators indicated they wanted to be able to visualize the entire state and zoom in on areas warranting further investigation. In response, the program embarked on an effort to develop interactive maps that empower the user to visualize gaps in access to advanced stroke care throughout the state.

## Data and Methods

In the surveys administered to the 8 stroke coordinators, we asked them to identify the frequency and type of stroke transfers that occurred 25% or more of the time. Coordinators were determined to be the most knowledgeable about the frequency and types of transfers because of their work in stroke patient care management. We obtained hospital addresses for 1) hospitals that were transferring patients at least 25% of the time to a specific tertiary hospital and 2) the tertiary hospitals that were receiving those patients. For the purposes of our maps, we classified the former as spokes and the latter as hubs. We then used the certification databases of Det Norske Veritas (DNV) (8) and The Joint Commission (9) to identify the level of stroke care certification for each hospital. We classified each hospital as a comprehensive stroke center, a primary stroke center, or an acute stroke–ready hospital. Stroke certification designations indicate the level of care a hospital may provide to stroke patients. We geocoded hospital addresses in ArcMap version 10.7 (Esri). We used Esri’s Network Analyst to calculate drive-time estimates and depicted these as “as the crow flies” lines to avoid confusing drive paths. The analytic assumptions for the calculation of estimated drive times were 1) time in minutes, 2) length in miles, 3) U-turns at junctions allowed, and 4) output shapes as straight lines. Then, we used Esri’s Network Analyst extension, Origin Destination Cost Matrix, and US Detailed Streets in Smart Data Compression to create transfer lines between hospitals and estimate driving distances and ambulance transport times. To generate interactive maps, we imported the map layers to Tableau version 2021.4.3 (Tableau Software, LLC) as a geodatabase. Finally, we used “embed” code to publish 2 interactive dashboards online (www.dhs.wisconsin.gov/coverdell/stroke-transfer-map.htm) so users could view maps for the entire state and by hospital. The maps can be accessed on a desktop computer, mobile device, or tablet. We completed user testing of 2 interactive maps and promotion to the program’s statewide partners in April 2022. We announced the maps via email to hospitals, emergency management services, and partner distribution lists and at our quarterly partner meetings.

## Highlights

The stroke transfer maps make it possible to clearly visualize the distances and times between hospitals by stroke certification status and identify the gaps in stroke systems of care. Overall, the median driving distance between small rural hospitals and larger tertiary hospitals was 44.5 (IQR, 29.0–73.3) miles, and the median transport time was 52.5 (IQR, 33.8–86.0) minutes. The maps also show geographic patterns in locations of hospitals and transfer patterns by hospital.

## Action

The Wisconsin Coverdell Stroke Program drew on its longstanding partnerships with regional stroke coordinators to develop a functional mapping approach. Not only do the interactive maps respond to the needs of stroke partners but they also help the state program understand differences in hospital certifications, geography, and resources, and take action to improve stroke care. Furthermore, interactive stroke transfer maps may serve as a model for other chronic disease programs looking to identify gaps in systems of care. In addition to receiving sizeable traffic to the online dashboards from other states, the Wisconsin Coverdell Stroke Program has provided direct technical assistance to health department staff from other states looking to develop interactive maps through their involvement in the Chronic Disease GIS Network (7).

Besides supporting the work of health departments, interactive stroke transfer maps can inform hospital administrators’ understanding of service capacity by highlighting the catchment area for each hospital. These maps can also draw policymakers’ attention to areas that might benefit from telemedicine policies that help address unequal coverage of services, resources, and facilities in rural areas, or transport-bypass protocols that help community and emergency services personnel best route their stroke patients to appropriate centers of care. Future versions of the Wisconsin Coverdell Stroke Program maps may incorporate telemedicine consultations, and, because some stroke transfers occur by air, aerial stroke transfers.
